# Supplementary material for: Estimating the cumulative incidence of COVID-19 in the United States using influenza surveillance, virologic testing, and mortality data: Four complementary approaches
Source: PLoS Comput Biol. 2021 Jun 17;17(6):e1008994. doi: 10.1371/journal.pcbi.1008994 (PMC8241061; doi:10.1371/journal.pcbi.1008994)

Divergence by Location

Figs A and B show the *Divergence* approach model fits for all available locations. COVID-19 is treated as an intervention, and we measure COVID-19 impact on observed CDC ILI, using IDEA model predicted ILI, virology predicted ILI, and historical projection predicted ILI as counterfactuals. The difference between the higher observed CDC ILI and the lower predicted ILI is the measured impact of COVID-19. The impact directly maps to an estimate of COVID-19 ILI-symptomatic case counts. Virology-predicted ILI is omitted when virology data is not available. We note that model fit quality varies by location. CDC reported ILI activity is plotted in blue, historical projection predicted ILI is plotted in purple, IDEA model predicted ILI is plotted in orange, and virology predicted ILI is plotted in green. We note that this approach is meaningful only at the beginning of the outbreak (March 2020), while ILI surveillance systems are still fully operational and before they are impacted by COVID-19. The disappearance of the divergence does not mean that the outbreak is over, but rather that the ILI signal is no longer reliable. As a reference, Figs C and D show the model fits for the same locations during the COVID-free 2018-2019 flu season.

Figure A: Divergence model fits for first half of locations.

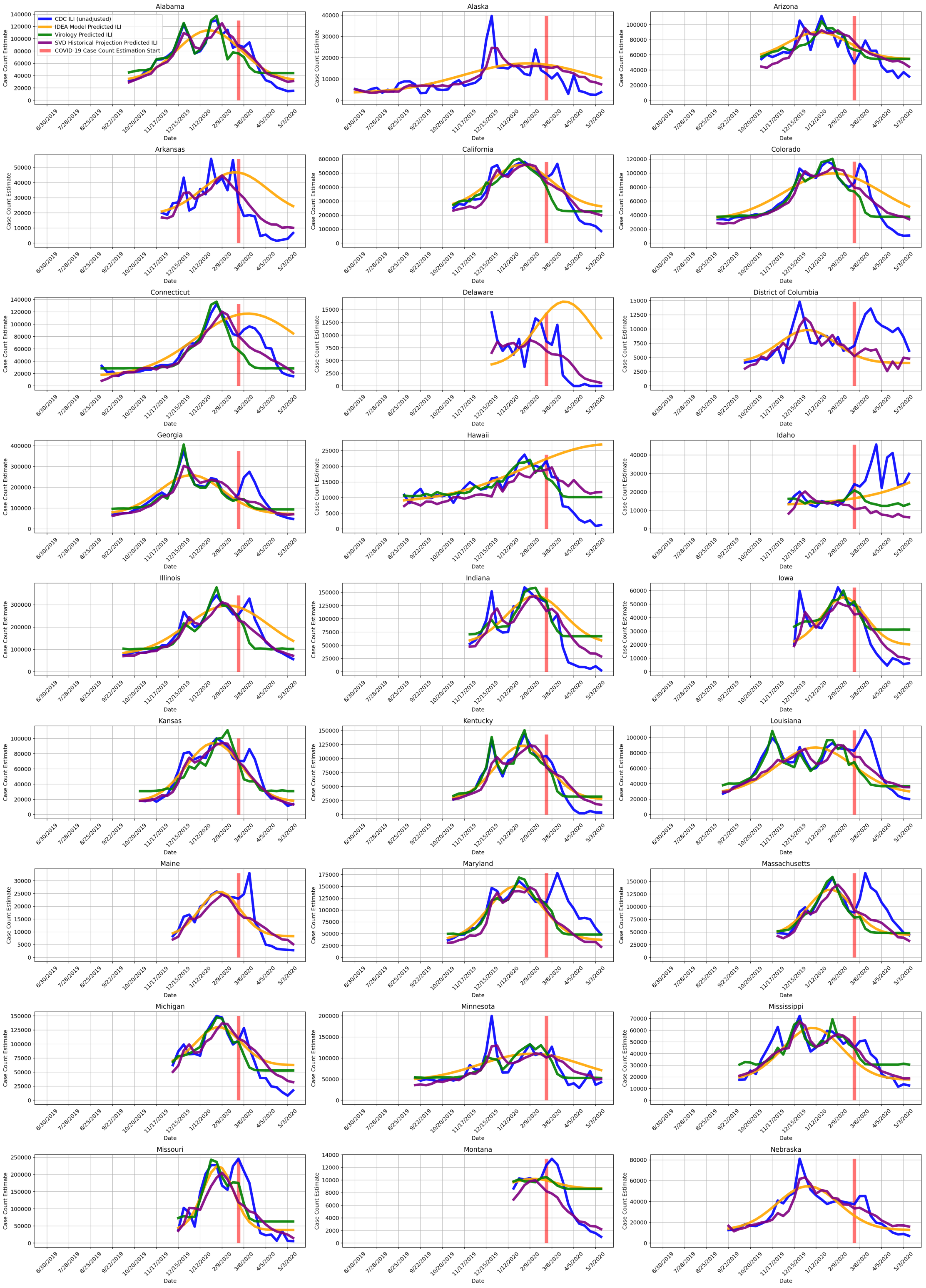

Figure B: Divergence model fits for second half of locations.

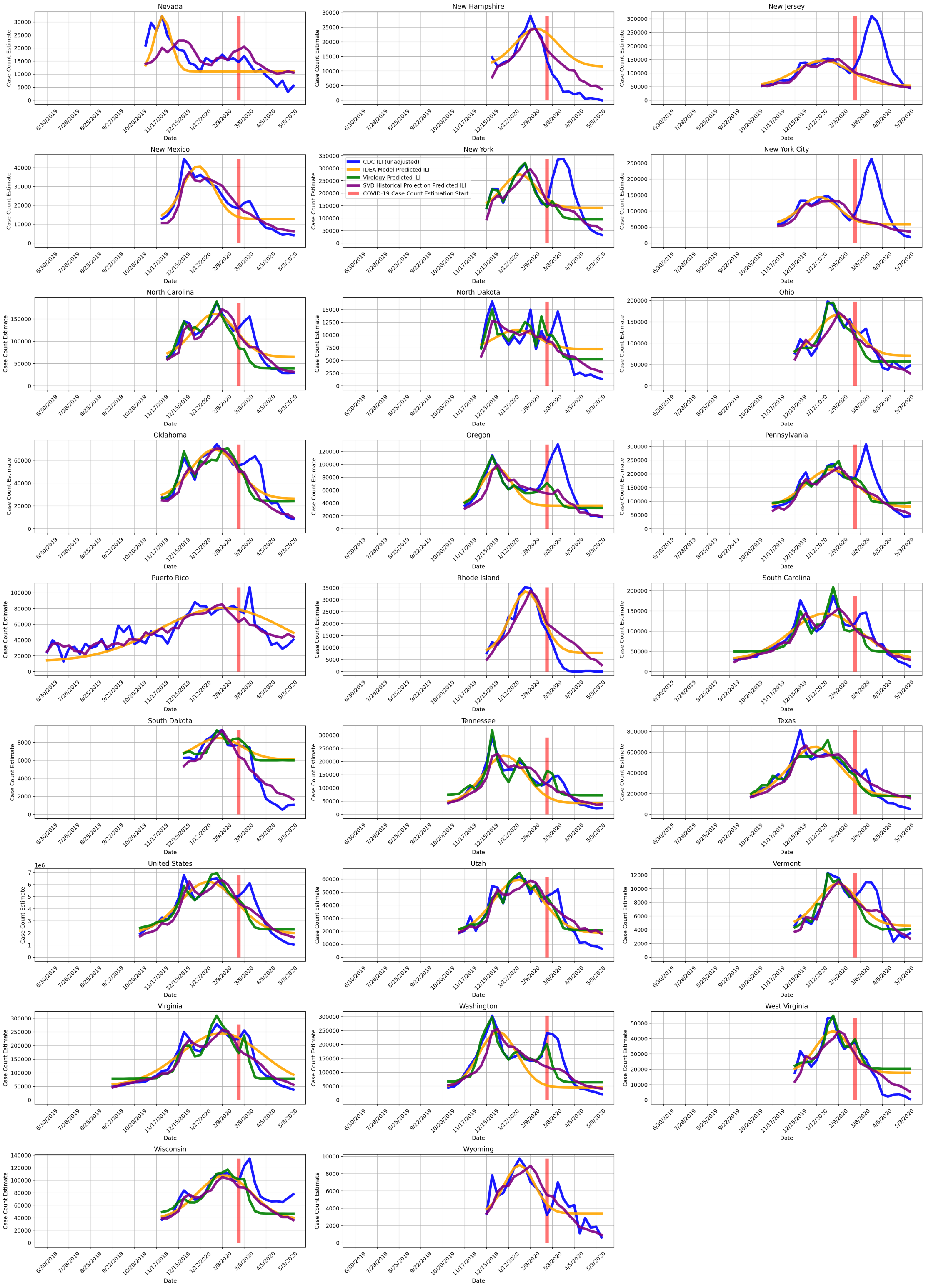

Figure C: ILI model fits during the COVID-free 2018-2019 flu season for the first half of locations.

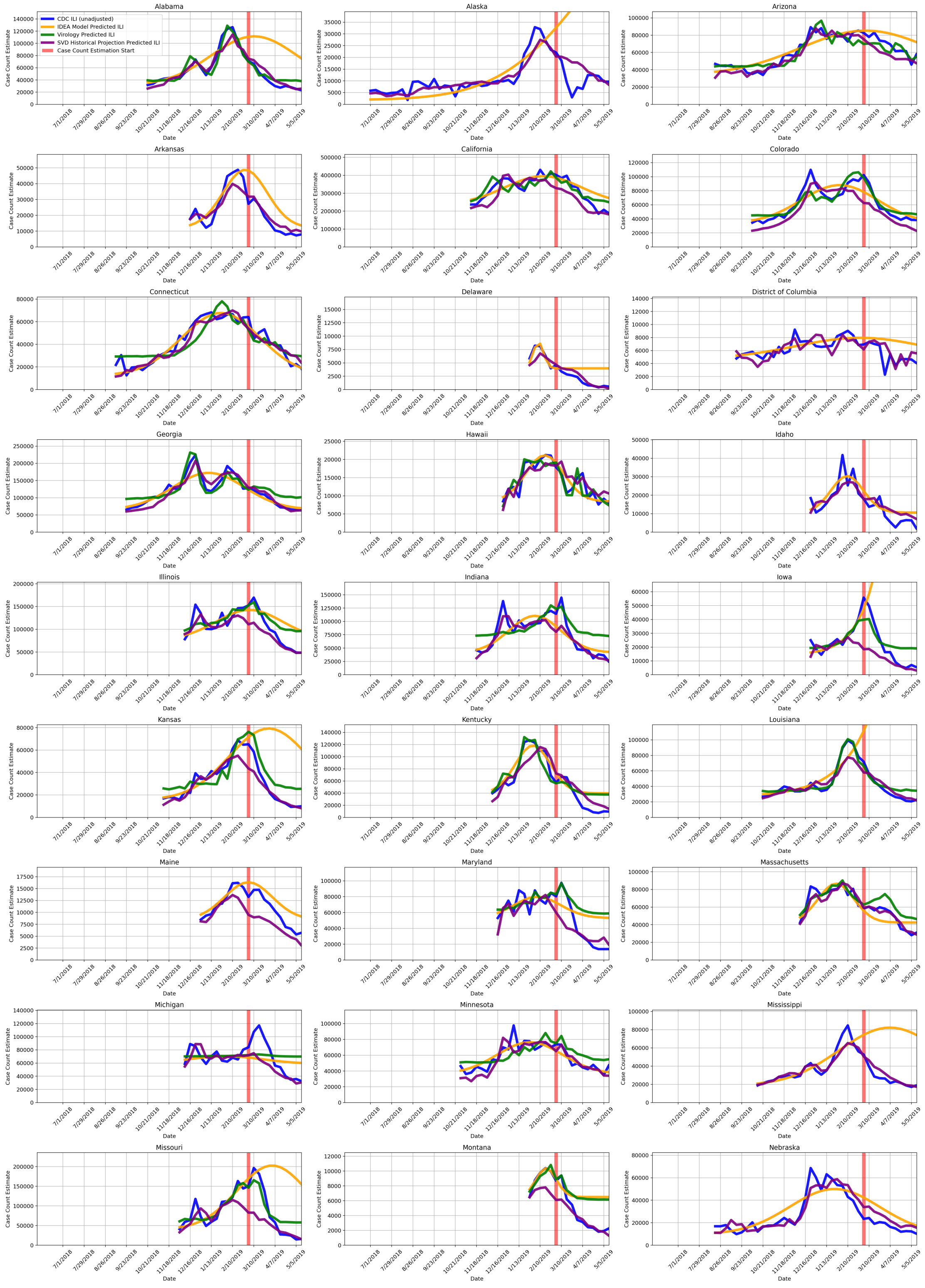

Figure D: ILI model fits during the COVID-free 2018-2019 flu season for the second half of locations.

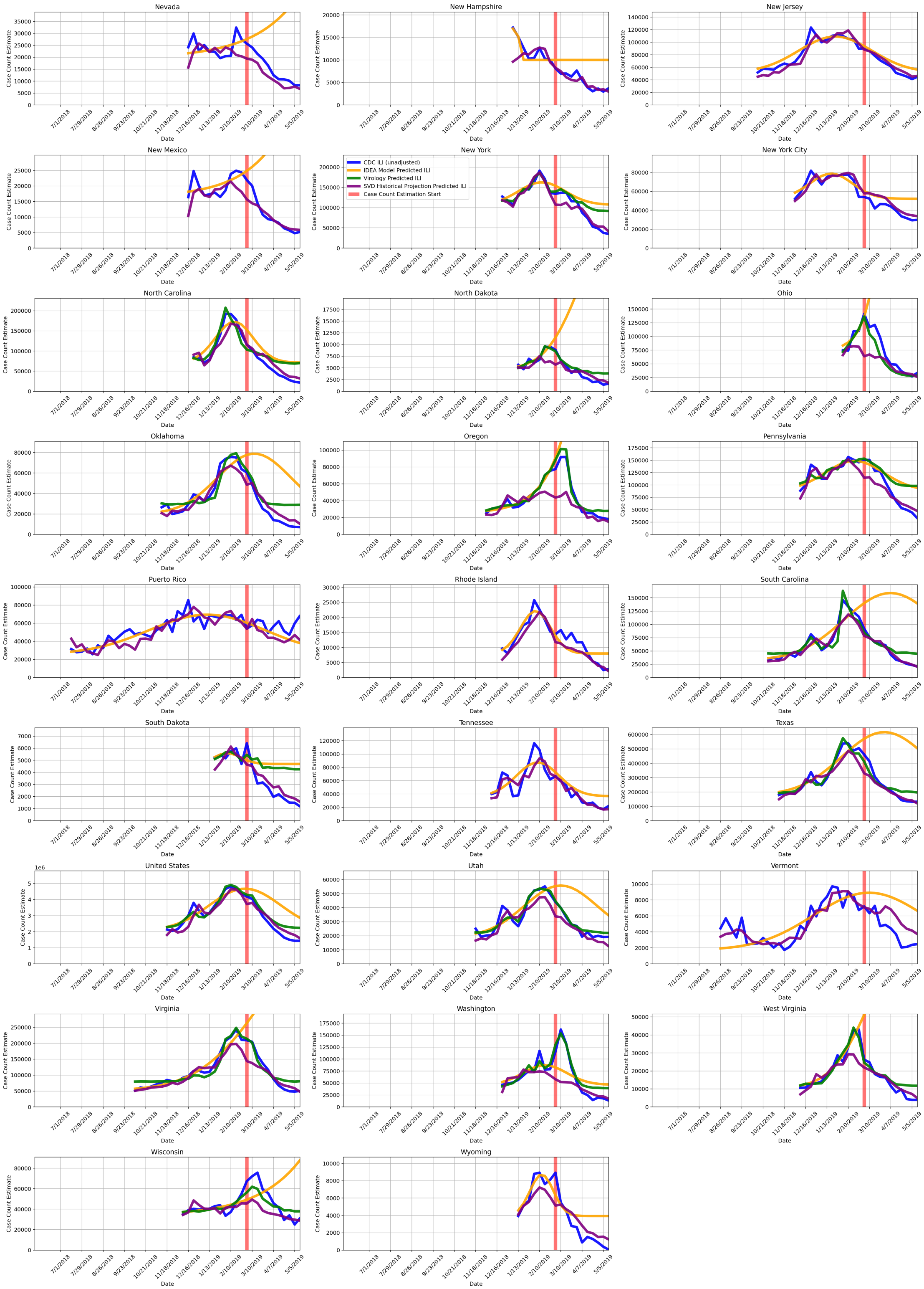

Supplement: S1 Fig — Figures A and B in S1 Fig show the Divergence approach model fits for all available locations. COVID-19 is treated as an intervention, and we measure COVID-19 impact on observed CDC ILI, using IDEA model predicted ILI, virology predicted ILI, and historical projection predicted ILI as counterfactuals. The difference between the higher observed CDC ILI and the lower predicted ILI is the measured impact of COVID-19. The impact directly maps to an estimate of COVID-19 ILI-symptomatic case counts. Virology-predicted ILI is omitted when virology data is not available. We note that model fit quality varies by location. CDC reported ILI activity is plotted in blue, historical projection predicted ILI is plotted in purple, IDEA model predicted ILI is plotted in orange, and virology predicted ILI is plotted in green. We note that this approach is meaningful only at the beginning of the outbreak (March 2020), while ILI surveillance systems are still fully operational and before they are impacted by COVID-19. The disappearance of the divergence does not mean that the outbreak is over, but rather that the ILI signal is no longer reliable. As a reference, Figures C and D in S1 Fig show the model fits for the same locations during the COVID-free 2018–2019 flu season. (PDF) [file pcbi.1008994.s001.pdf]
