# Supplementary material for: Estimating the cumulative incidence of COVID-19 in the United States using influenza surveillance, virologic testing, and mortality data: Four complementary approaches
Source: PLoS Comput Biol. 2021 Jun 17;17(6):e1008994. doi: 10.1371/journal.pcbi.1008994 (PMC8241061; doi:10.1371/journal.pcbi.1008994)

Time Series Plots for All Methods

Figs A and B show the cumulative estimated counts for each week over the entire study period of March 1, 2020 to May 16, 2020, compared with cumulative reported counts, in each location in the United States. The solid and dotted lines indicate adjusted and unadjusted methods, respectively. Due to the seasonal nature of ILI information, estimates from all methods besides $mMAP$ are limited to April 4, 2020.

Figure A: Cumulative case time series for first half of locations.

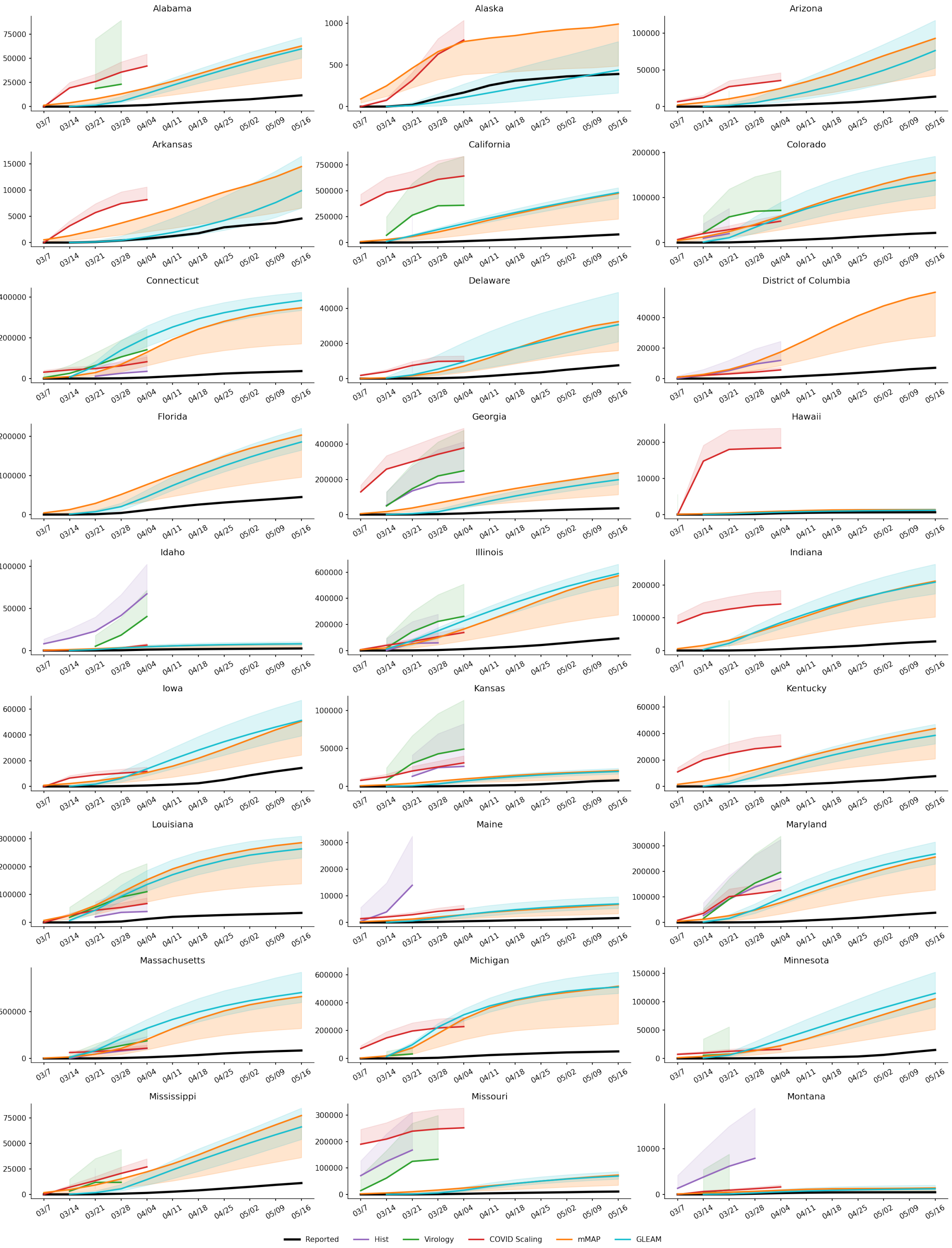

Figure B: Cumulative case time series for second half of locations.

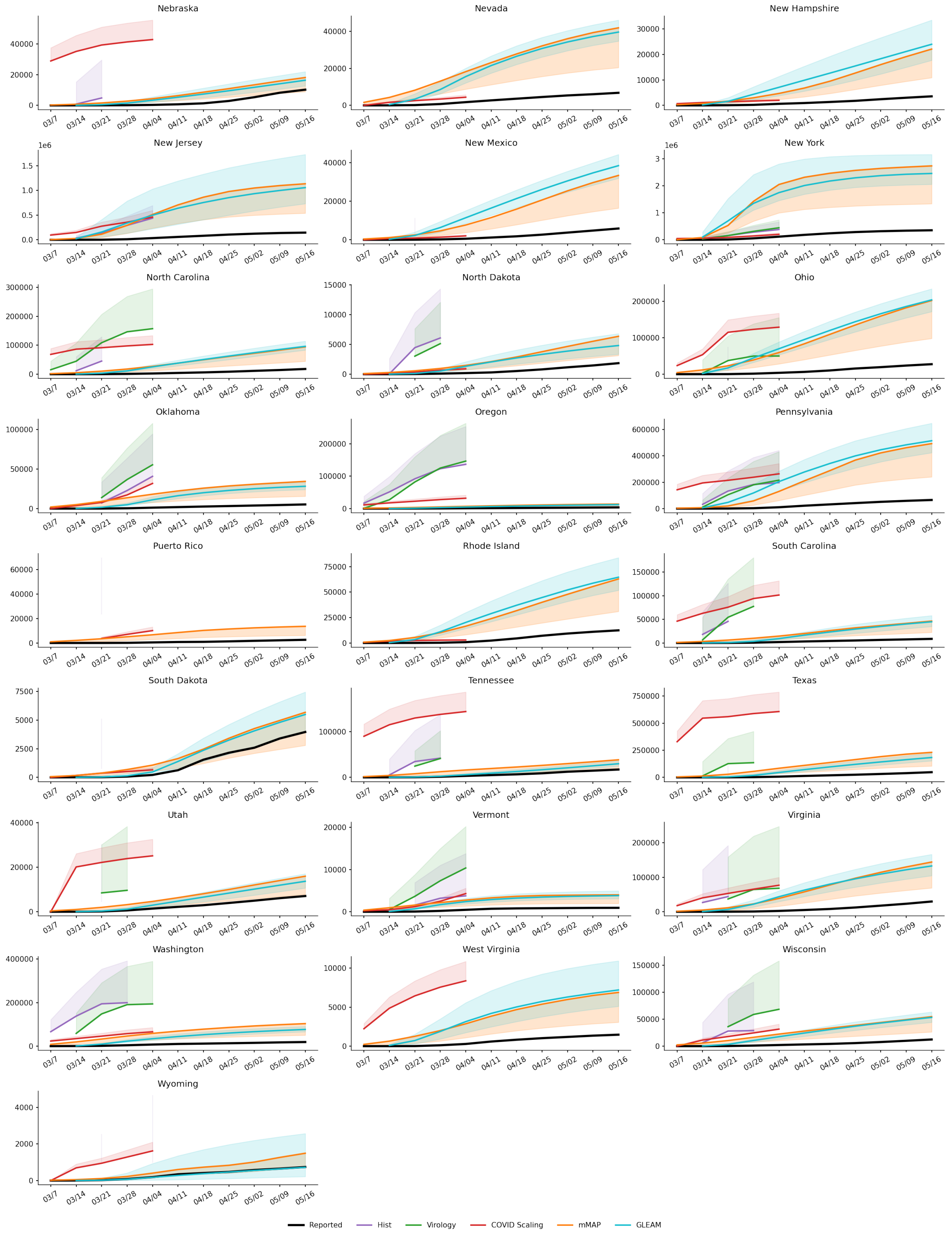

Supplement: S2 Fig — Figures A and B in S2 Fig show the cumulative estimated counts for each week over the entire study period of March 1, 2020 to May 16, 2020, compared with cumulative reported counts, in each location in the United States. The solid and dotted lines indicate adjusted and unadjusted methods, respectively. Due to the seasonal nature of ILI information, estimates from all approaches besides mMAP and GLEAM are limited to April 4, 2020. (PDF) [file pcbi.1008994.s002.pdf]
