# Supplementary material for: Estimating the cumulative incidence of COVID-19 in the United States using influenza surveillance, virologic testing, and mortality data: Four complementary approaches
Source: PLoS Comput Biol. 2021 Jun 17;17(6):e1008994. doi: 10.1371/journal.pcbi.1008994 (PMC8241061; doi:10.1371/journal.pcbi.1008994)
Supplement: S1 Text — We explore an additional model-based method for ILI counterfactual estimation for the Divergence approach. (PDF) [file pcbi.1008994.s003.pdf]

A Third Divergence Method: Incidence Decay and Exponential Adjustment Model

The Incidence Decay and Exponential Adjustment (IDEA) model [1] is a single equation epidemiological model that estimates disease incidence over time early in an outbreak while accounting for control activities and behaviours. The model is as follows:

$$I(t) = \left(\frac{R_0}{(1+d)^t} \right)^t$$

where $I(t)$ is the incident case count at serial interval time step t. R_0 is the basic reproduction number, and d is a discount factor modeling reductions in the effective reproduction number with time due to public health interventions, changes in public behavior, environmental factors, and the depletion of susceptible hosts. The IDEA model has been shown to be identical to Farr's law for epidemic forecasting and can be expressed in terms of a susceptible-infectious-removed (SIR) compartmental model with improving control [2].

We can fit the IDEA model to ILI case counts from the start of the 2019-2020 influenza season to the last week of February 2020. The start of the 2019-2020 influenza season is defined in a location specific manner as the first occurrence of two consecutive weeks with ILI activity above 2%. Model fitting is done using non-linear least squares with the Trust Region Reflective algorithm as the optimizer. Next, the model is used to predict what ILI would have been had the COVID-19 pandemic not occurred. In other words, we use the IDEA model ILI estimates as the counterfactual when assessing the impact of the COVID-19 intervention. When fitting the IDEA model, we use a serial interval of half a week, consistent with the serial interval estimates from [3] for influenza. We note that serial interval estimates from [4] for COVID-19 as well as from [5] for SARS-CoV-1 are longer than that of influenza, but that is not an issue as we use IDEA to model ILI.

References

1. Fisman DN, Hauck TS, Tuite AR, Greer AL. An IDEA for short term outbreak projection: nearcasting using the basic reproduction number. PloS one. 2013;8(12).

2. Santillana M, Tuite A, Nasserie T, Fine P, Champredon D, Chindelevitch L, et al. Relatedness of the incidence decay with exponential adjustment (IDEA) model,"Farr's law" and SIR compartmental difference equation models. Infectious disease modelling. 2018;3:1–12.

3. Vink MA, Bootsma MCJ, Wallinga J. Serial Intervals of Respiratory Infectious Diseases: A Systematic Review and Analysis. American Journal of Epidemiology. 2014;180(9):865–875. doi:10.1093/aje/kwu209.

4. Li Q, Guan X, Wu P, Wang X, Zhou L, Tong Y, et al. Early transmission dynamics in Wuhan, China, of novel coronavirus–infected pneumonia. New England Journal of Medicine. 2020;.

5. Lipsitch M, Cohen T, Cooper B, Robins JM, Ma S, James L, et al. Transmission dynamics and control of severe acute respiratory syndrome. Science. 2003;300(5627):1966–1970.
