# Supplementary material for: Estimating the cumulative incidence of COVID-19 in the United States using influenza surveillance, virologic testing, and mortality data: Four complementary approaches
Source: PLoS Comput Biol. 2021 Jun 17;17(6):e1008994. doi: 10.1371/journal.pcbi.1008994 (PMC8241061; doi:10.1371/journal.pcbi.1008994)
Supplement: S2 Text — Theoretical backing for virology-based estimation. (PDF) [file pcbi.1008994.s004.pdf]

Virology-based Estimation

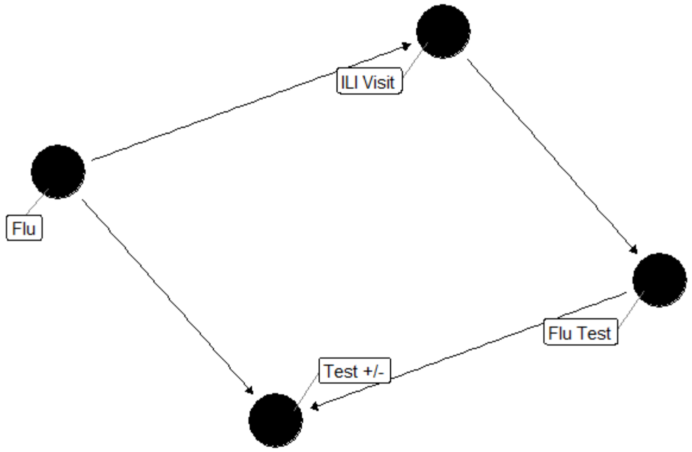

Figure A: Causal DAG affecting flu positive results.

Both the virology-based *Divergence* model and the *COVID Scaling* method rely on the extrapolation of positive testing data to the actual symptomatic incidence of the disease. The causal diagram shown in Fig A shows that an individual's flu test result depends on whether they have the disease, but also whether they receive a test in the first place (by going through the ILI visit path). More broadly, the relationship between test positive results and true disease counts are influenced by testing availability. We approximate the availability using the total administered tests divided by ILI cases. Identical reasoning applies for analysis of COVID-19 cases, as done in the COVID Scaling method.

We formulate a valid control as having the following two properties:

1. The control produces a reliable estimate of ILI activity.
2. The control is not affected by the COVID-19 intervention (that is, the model of ILI conditional on any relevant predictors is independent of COVID-19).

In Table A, we show that the total positive tests divided by the availability satisfies both properties and successfully estimates the true flu counts (in the perfectly distributed case) even when a surge of COVID-19 cases is added.

	Baseline cases			With COVID-19 cases		
Data	1	2	3	1	2	3
Flu (F)	*20*	*20*	*40*	*20*	*20*	*20*
ILI (I)	100	100	200	200	200	400
Test (N)	10	50	50	10	50	50
Positive (F^+)	2	10	10	1	5	2.5
Availability (N/I)	0.1	0.5	0.25	0.05	0.25	0.125
**Predict** $\hat{F}$	**20**	**20**	**40**	**20**	**20**	**20**
**Predict** $\hat{I}$	**100**	**100**	**200**	**100**	**100**	**100**

Table A: Series of examples showing that the proposed estimator ($F^+ \cdot I/N$) predicts flu cases correctly even when potential COVID-19 is added. $\hat{I} = f(\hat{F})$ is modeled as an affine function $\alpha + \beta\hat{F}$ learned over pre-COVID-19 data (here with $\beta = 5$ for simplicity).
