# Supplementary material for: Estimating the cumulative incidence of COVID-19 in the United States using influenza surveillance, virologic testing, and mortality data: Four complementary approaches
Source: PLoS Comput Biol. 2021 Jun 17;17(6):e1008994. doi: 10.1371/journal.pcbi.1008994 (PMC8241061; doi:10.1371/journal.pcbi.1008994)
Supplement: S3 Text — Sensitivity analysis on different assumptions of COVID Scaling. (PDF) [file pcbi.1008994.s005.pdf]

COVID Scaling Sensitivity Analysis

The COVID Scaling method estimates the probability p_{jt} that a given ILI patient in state j and week t has COVID-19. Although we account for sparse data, testing backlogs, and false negatives, we must assume that testing is applied uniformly conditional on observable data. That is, the probability a patient is tested for COVID-19, conditional on the observed symptoms, is independent of whether they have COVID-19 or a different illness. This assumption can break, for example when health workers at a nursing home outbreak are preemptively tested. Because this form of confounding is difficult to measure, we conduct a sensitivity analysis.

The most likely failure of this assumption is that testing is biased towards patients more likely to have COVID-19. Let C denote having COVID-19 and T denote being tested. Let $p_{true} = p_{jt} = P(C)$ and p_{obs} be the posterior estimate from the COVID Scaling method. Since

$$p_{obs} = P(C|T) = \frac{P(T|C)P(C)}{P(T|C)P(C) + P(T|C^c)P(C^c)}$$

We model $P(T|C) = \alpha P(T|C^c)$, where $\alpha > 1$, with an extreme of $\alpha = 1.5$, meaning that the COVID-19 infected are 50% more likely than non-infected to get tested. Then

$$p_{obs} = \frac{\alpha P(T|C^c)p_{true}}{\alpha P(T|C^c)p_{true} + P(T|C^c)(1 - p_{true})}$$

yielding

$$p_{true} = \frac{p_{obs}}{\alpha - \alpha p_{obs} + p_{obs}}$$

Finally, in each state, we vary α linearly with testing availability, defined by number of COVID-19 tests over total ILI patients for each week, such that the minimum availability receives $\alpha = 1.5$ and maximum availability receives $\alpha = 1.0$. This gives adjusted probabilities, which we use to recompute COVID-19 projections. The ratios of cases after this adjustment to the original projected cases are shown in Table A.

Max α	25%	50%	75%
1.5	0.78	0.81	0.88
1.25	0.87	0.89	0.93
1.10	0.94	0.95	0.97

Table A: The median and quartile scaling adjustments across states when we assume varying levels of α (the increased likelihood for COVID-19 positive patients to get tested). For example, in the most extreme α, the median state had an estimated case count of 0.81 times the original estimate from COVID Scaling.
