# Supplementary material for: Estimating the cumulative incidence of COVID-19 in the United States using influenza surveillance, virologic testing, and mortality data: Four complementary approaches
Source: PLoS Comput Biol. 2021 Jun 17;17(6):e1008994. doi: 10.1371/journal.pcbi.1008994 (PMC8241061; doi:10.1371/journal.pcbi.1008994)

Mortality-MAP Analysis

1 Mortality-Mapping and Richardson-Lucy Deconvolution

Predicting cases from observed deaths can be framed as a deconvolution problem, where the observations (COVID-19 deaths) are convoluted signals of underlying parameters (COVID-19 cases that result in death) with known transition probabilities between the two states (the distribution of time from COVID-19 symptom onset to death). There has been research on deconvolution in signal processing, and our work, along with the deconvolution research for the 1918 flu [1], relies on research on deconvolution for Positron Emission Tomography [2]. In [2], it is shown that a Richardson-Lucy (RL) algorithm, or expectation maximization (EM) algorithm, converges to the maximum likelihood estimate of the unknown parameters. It can be shown that each iteration for *mMAP* is the same as the EM step presented in [2]. First note that each iteration step (ignoring the right-censoring for now) in *mMAP* is

$$C_{d^*}^{(i)}(t) = \sum_{\tau=t+1}^{t_{max}} D(\tau) \cdot \frac{p(T=(\tau-t)) \cdot \frac{C_d^{(i-1)}(t)}{\sum C_d^{(i-1)}(t)}}{\sum\limits_{s=1}^{\tau-1} p(T=(\tau-s)) \cdot \frac{C_d^{(i-1)}(s)}{\sum C_d^{(i-1)}(t)}} \tag{1}$$

$$= C_d^{(i-1)}(t) \sum_{\tau=t+1}^{t_{max}} \frac{D(\tau) \cdot p(T=(\tau-t))}{\sum\limits_{s=1}^{\tau-1} p(T=(\tau-s)) \cdot C_d^{(i-1)}(s)} \tag{2}$$

[2] discusses this problem in the context of reconstructing positron emission tomography (PET) images. They denote $n^*(d)$ as the observed count of data in tube d, $\lambda(b)$ as the unobserved count of data in box b, and $p(b, d)$ as the probability of transmission from B to tube d. They demonstrate that the expectation-maximization algorithm to solve the maximum likelihood estimate for the $\lambda(b)$'s involves this step:

$$\lambda^{new}(b) = \lambda^{old}(b) \sum_{d=1}^{D} \frac{n^*(d)p(b,d)}{\sum_{b'=1}^{B} \lambda^{old}(b')p(b',d)} \tag{3}$$

where D and B are the total numbers of tubes and boxes, respectively. Note that this step is the same as step (2) in *mMAP*, replacing $n^*(d)$ with observed deaths $D(\tau)$, $\lambda(b)$ with unobserved cases $C(t)$, and $p(b, d)$ with the transition probabilities $p(T = (\tau - t))$. Therefore, while both methods are presented differently, they rely on the same iterative process. The mathematical justifications provided for RL deconvolution in [2], specifically that the likelihood monotonically increases and that it is concave (so EM converges to a global maximum), can therefore be used to justify *mMAP*.

2 Simulated and Empirical Validation

To validate *mMAP*, it was analyzed using simulated and real death data until June 7 from six countries: United States, Italy, Spain, Germany, Japan and South Korea. Fig A compares *mMAP* predicted cases with reported cases. To visually scale the reported cases, the following equation is used:

$$\textit{reported-scaled} = \textit{reported} \cdot \frac{\sum \textit{predicted}}{\sum \textit{reported}}$$

While the scales differ, the trends of predicted cases generally follow the trends of reported cases. The later increase of reported cases as opposed to predicted cases in most countries (except Germany) could be a result of increasing case detection around the start of April. As testing increases, we would expect to see more of a relative increase in reported cases than in reported deaths (because we would likely be picking up more of the less severe cases), which would cause the reported cases to increase more steeply than *mMAP* predictions.

In Fig B, the deaths for each country are simulated from the reported cases. Deaths are stochastically simulated from the reported cases using an sCFR of 0.013 and the same distribution from symptom onset to death used in the rest of this paper [3]. From the simulated deaths, *mMAP* predicts the original cases. This figure demonstrates that *mMAP* recovers the original cases reasonably well.

Both plots offer validation that *mMAP* can successfully predict the trend of the reported cases. However, these plots do not demonstrate if the scale of *mMAP* predictions are on target, as this is influenced by the under-reporting of deaths and the sCFR.

References

1. Goldstein E, Dushoff J, Ma J, Plotkin JB, Earn DJ, Lipsitch M. Reconstructing influenza incidence by deconvolution of daily mortality time series. Proceedings of the National Academy of Sciences. 2009;106(51):21825–21829.

2. Shepp LA, Vardi Y. Maximum likelihood reconstruction for emission tomography. IEEE transactions on medical imaging. 1982;1(2):113–122.

3. Linton NM, Kobayashi T, Yang Y, Hayashi K, Akhmetzhanov AR, Jung Sm, et al. Incubation period and other epidemiological characteristics of 2019 novel coronavirus infections with right truncation: a statistical analysis of publicly available case data. Journal of clinical medicine. 2020;9(2):538.

Figure A: *mMAP* predictions compared to reported cases.

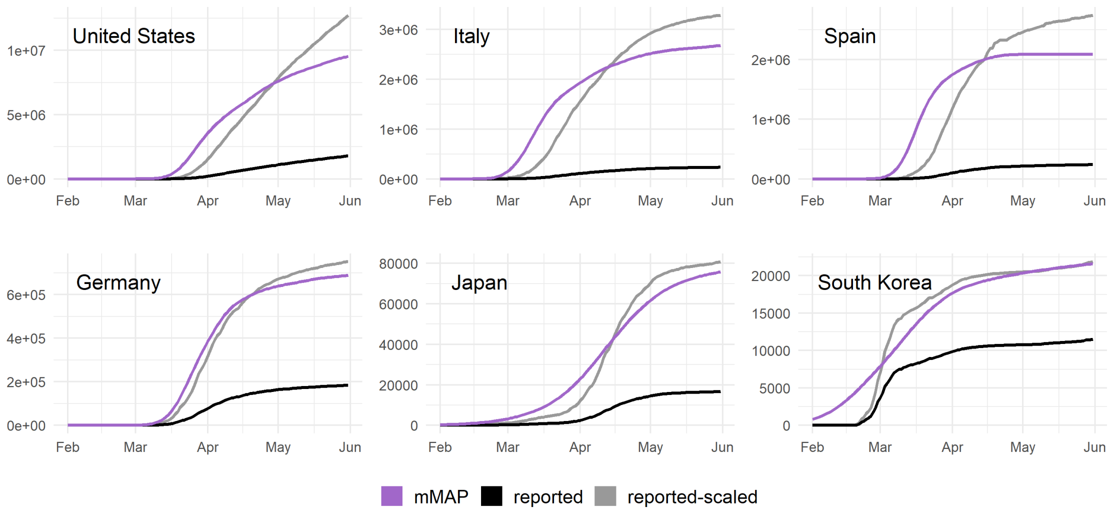

Figure B: Simulated *mMAP* predictions compared to reported cases.

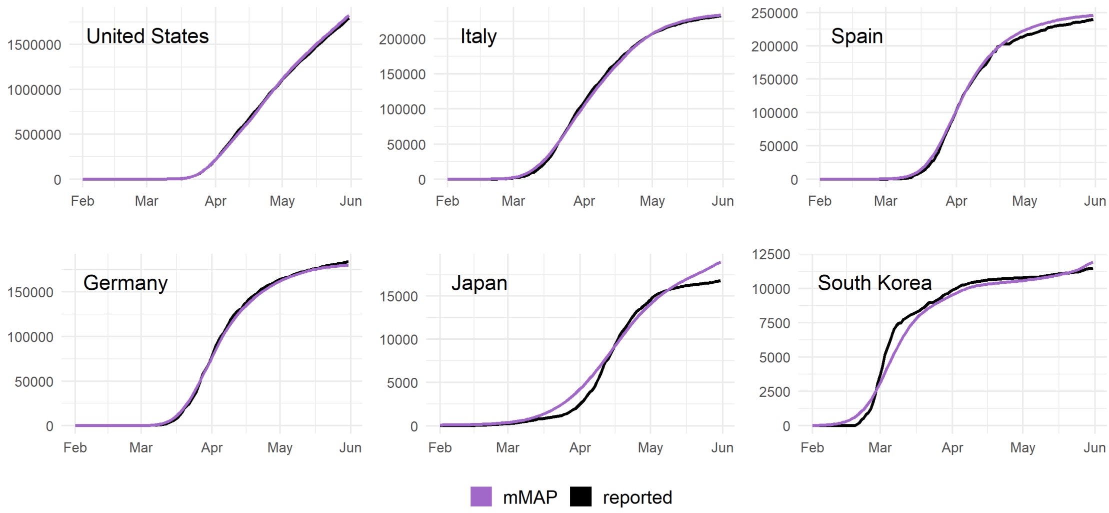

Supplement: S4 Text — Theoretical backing for Mortality-MAP method. (PDF) [file pcbi.1008994.s006.pdf]
