# Supplementary material for: Estimating the cumulative incidence of COVID-19 in the United States using influenza surveillance, virologic testing, and mortality data: Four complementary approaches
Source: PLoS Comput Biol. 2021 Jun 17;17(6):e1008994. doi: 10.1371/journal.pcbi.1008994 (PMC8241061; doi:10.1371/journal.pcbi.1008994)
Supplement: Attachment — Submitted filename: responses_to_reviewers.pdf [file pcbi.1008994.s007.pdf]

Point by point response to reviewers

Thank you for reviewing our work and for the opportunity to revise our manuscript following your suggestions. We believe our results have strengthened due to the feedback. Please find below a point by point response. Changes and revisions have been highlighted in blue in the marked manuscript copy. We have also added an approach named GLEAM. Information on this approach and our reason for its addition is provided at the end of this document, as well as the Methods section.

Reviewer #1:

I appreciate the opportunity to review "Estimating the Early Outbreak Cumulative Incidence of COVID-19 in the United States: Three Complementary Approaches" by Lu et al. The authors utilize influenza-like illness surveillance data, COVID testing volume, and reported COVID deaths to estimate cumulative symptomatic COVID cases in US states. Though I think this work is important and that the manuscript well-written, I have a few concerns regarding the authors' methodology. Please see my comments below.

Results

Line 64: I recommend explicitly mentioning that the "Divergence" and "COVID Scaling" approaches estimate symptomatic COVID cases up to the week of April 4th and that the "mMaP" approach estimates cases up to May 16th.

Response: We appreciate the reviewer's request to state clearly the dates of estimates for each of our methods. In the revised version of our manuscript we have updated the opening sentence of the results section to make this point more clear:

> *"We implement four approaches - Divergence, COVID Scaling, mMAP, and GLEAM - to estimate the cumulative symptomatic incidence of COVID-19 within the US from March 1 to April 4, 2020 (we further extend mMAP and GLEAM predictions to May 16, 2020)"*

Line 95: It's briefly touched on in the discussion but I think it's important to acknowledge that there was a "worried well" spike in ILI visits at general practitioner offices at the beginning of the epidemic in the US (early March) and then a sharp decline in ILI due to stay-at-home orders, increased use of tele-health services, seeking healthcare at providers that are not included in ILI surveillance (e.g., urgent care, emergency rooms), and the end of the flu season.

Response: The reviewer's suggestion is important. In the revised version of our manuscript we have made modifications to incorporate these points, in the Limitations section of the Discussion:

"...these estimates are likely to change over time, especially during the course of a pandemic. At the beginning of the pandemic, many more than usual may have paid their doctors a visit on the first sign of any ILI symptoms. Moreover, the weekly symptomatic incidence estimates from this

method decrease towards the end of March, perhaps caused by a drop in health care seeking behavior after the declaration of a national emergency on March 13, 2020 and the widespread implementation of shelter-in-place mitigation strategies which may have increased the use of medical services and health providers that are not included in the ILI surveillance network, such as telehealth services and urgent care."

Discussion

Line 232: ILI surveillance does not likely accurately capture ILI dynamics for elderly individuals residing in nursing homes (the individuals most at risk for severe COVID and death).

Response: Thank you, we have reported this important fact under Limitations in the Discussion section:

"Furthermore, we note that ILI surveillance networks may not always accurately measure ILI for the most at risk elderly individuals who reside in nursing homes."

Methods

General comment: I recommend including uncertainty estimates for ILI projections, which would in turn enable uncertainty estimates for COVID burden for the approaches that utilize ILI data.

Response: We agree that providing uncertainty estimates for ILI projections is important. Our ILI-based methods only return point estimates (although we have added an additional method, GLEAM, that does have uncertainty estimates). However, even though the methods at a given parameter value returns a point estimate, we are able to vary the underlying parameter values based on their uncertainty ranges. As a result, we obtained a range of estimates for each method, labeled adjusted vs unadjusted estimates, which can be interpreted as uncertainty bookends. The importance of acknowledging the high degree of uncertainty using a single approach was an important motivator for us to pursue producing estimates using multiple approaches based on disparate data sources. We hope this will provide a clearer idea of the uncertainty in our projections.

Line 324: The IDEA model/Farr's Law seems too simplistic for projecting ILI cases in the absence of COVID. Did the authors assess whether this method accurately captures ILI trajectories in prior seasons? It seems more appropriate to use a time series model fit to historical ILI data from several past seasons (state-level data are available going back to 2010-2011), which can account for seasonality in each state and provide prediction intervals for ILI projections.

Response: We appreciate the reviewer's suggestion. We found that the IDEA model had mixed prediction performance in previous seasons, sometimes working well and sometimes not

working well. We chose to keep this method in our manuscript for two reasons, (1) this approach makes different assumptions than our other methods, and (2) we suspect that the shortcomings of this method are uncorrelated with our other methods. To better allow the reader to gain perspective on when this method works and when it does not, we have included ILI model fits during the COVID-free 2018-2019 flu season in the Supplementary Materials.

Given that the IDEA-based estimates had such mixed results, we included an additional time series-based method designed to exploit historical ILI data from past seasons to provide prediction estimates, as suggested by the reviewer. To inform our choice of method, we compared two time series models, one based on SARIMA models, and one based on reconstructing ILI activity based only on the most salient features of past seasons. The latter method uses past seasons information and a dimensionality reduction approach based on the Singular Value Decomposition (SVD) to calculate a small set of basis vectors that are capable of reconstructing the most salient features of the past observed flu seasons. These basis vectors are then used to project the likely trajectory of a future (partially unobserved) flu season. Our experiments showed that the SVD-based projection method far outperformed the SARIMA approaches. Thus, we have added the SVD method to our main analysis and have moved IDEA to the Supplementary Materials. We believe this has strengthened our results and thank the reviewer for the excellent comment.

We have added the following text to the Methods section:

"Method 1: Singular Value Decomposition-based Historical Projection

Unseen future ILI can be projected by fitting a time series model to historical ILI data which can account for trends that capture state-specific seasonal trends. A simple approach capable of doing this could be a simple historical weekly average of past flu seasons; however this baseline approach would lack the flexibility to incorporate the thus-far observed season-specific patterns. Instead, in our approach, we model ILI during a specific flu season, in a specific location, as the historical average H at that location plus a season-specific component Y:

$$ILI = H + Y$$

We produce an estimate $\hat{Y}$ of the season-specific component Y by first arranging the historic ILI data into a matrix X with rows corresponding to weeks in a season (time) and columns corresponding to (space) observed ILI in all spatial locations in past seasons. We then compute the most salient features of this matrix (in other words, we identify the weeks with highest variance) by factorizing the matrix X using the singular value decomposition (SVD): $X = U\Sigma V^T$. The columns of U form an orthonormal basis for the ILI behavior during past and fully-observed seasons. The SVD algorithm returns these columns ranked by importance by the singular values in Σ. The estimate of the season-specific component is then computed using an elastic net regression using the (first elements of the) columns of $U\Sigma$ as predictors to fit the thus-far observed ILI activity as a response variable. The unseen portion of the season is then calculated using the full length of the columns U with the regression coefficients from the elastic net fit. In other words, we make the assumption that an epidemic year can be described as a linear combination of vectors using the historic data for all

locations. We scale U by the singular values in Σ so that elastic net's regularization will favor basis vectors with higher importance.

For each location, we use between 7 and 10 years of historical ILI data depending on data availability and quality.

We also perform variable selection by keeping only the basis vectors U_i where $\frac{\Sigma_{ii}}{trace(\Sigma)} > \frac{1}{52}$, yielding around 10 basis vectors kept depending on the year.

The elastic net regularization parameters are tuned by validation on a fraction of the current season data closest to the prediction period of interest. Figure 5 shows the improvement over a historical average during the 2018-2019 season prediction period by also incorporating a season specific component to model ILI. Overall, the SVD Historical Projection performs better than the historical average baseline. The locations for which SVD Historical Projection performs worse are locations where both methods have low error."

Line 369: I may have misunderstood but it seems that the authors multiplied % flu positive in 2020 by ILI counts from 2019. If this is the case, I recommend utilizing ILI data for 2010/2011 to 2018/2019 to compute an ILI "seasonal baseline" with 95% confidence intervals for each state, rather than applying 2019 ILI counts to 2020 dates.

Response: We apologize for the lack of clarity in our original submission. We did not multiply by counts from 2019, but rather we fit the regression parameters to data from the 2019-2020 season, prior to the COVID intervention. We have added the following text to clarify: *"we use 2019 pre-COVID-19 data to fit the regression."*

Line 392: Did the authors also explore how the decline in health-seeking behavior after stay-at-home measures potentially impacted p(visit|ILI)?

Response: We did find that there was a sharp decrease in the total number of reported patients in late March 2020 (stay-at-home orders were mostly enacted starting at the end of March / early April) without a larger than expected decrease in the number of providers. We did not attempt to adjust p(visit|ILI) to account for this or to extend our ILI-based predictions since we expected ILI-based methods to generally decrease in reliability outside of the regular flu season period. The irregularities in ILI reporting that usually occur outside of the flu season introduce confounders that would be hard to disentangle from the impact of stay-at-home measures.

Figures: Please define "unadjusted" and "adjusted" in figure captions.

Response: Thank you for suggesting this clarification. We have added the following to the figure captions:

“Adjusted methods take into account increased visit propensity (div-Hist, div-Vir, COVID Scaling) and excess influenza and pneumonia deaths along with a lower estimated case fatality rate (mMAP).”

Reviewer #2:

The authors present an analysis of COVID testing and ILI data to infer the true number of symptomatic COVID-19 cases in the US. This is of significant interest to researchers and policymakers. Additionally, using existing surveillance systems (such as ILI surveillance) is an important methodological approach that has been underused. However, I would recommend some revisions and updating the manuscript to reflect recent literature that estimates true infection rates, such as [1].

Major points:

1) Is the assumption of constant sCFR and IFR through time justified? I realize that the literature on IFR is constantly evolving, but increasing quality of care as well as changes in who is getting infected (e.g., nursing home outbreaks early in the pandemic) might have shifted the overall IFR (and thus sCFR) over time. Also, does adding uncertainty in the asymptomatic fraction substantially change the results? Given that uncertainty in IFR is incorporated, it seems odd to use a point estimate for the asymptomatic fraction, given that it has a substantial range associated with it (and, again, could be changing over time as the infected population changes). In any case, if these sources of uncertainty cannot be incorporated due to lack of data, I think it is important to at least address them in the discussion

Response: We appreciate the reviewer's comments about the IFR and sCFR. For the first point on the assumption of constant sCFR and IFR through time, it seems plausible that the IFR was decreasing in the early stages of the pandemic as we developed a better understanding of treatment for COVID-19. However, given the uncertainty in IFR estimates even for the entire pandemic, it is not yet possible to get a reliable estimate of the changing nature of IFR. As well, a report estimating the time-dependent IFR in Hubei province noted that the IFR estimate stabilized early on in the epidemic, towards the middle of February [1]. The IFR estimates from this paper, which are the IFR estimates still used by the CDC as of 10/27/2020, use data until the middle of April (April 14 to April 26 depending on the region). A more recent meta-analysis of all IFR estimates from serological studies until September 18 actually estimated a slightly higher national IFR once adjusting for age structure as was used in our study [2], which demonstrates the uncertainty around whether IFRs are decreasing or not, and another meta-analysis found no change in IFR over the first half of 2020 [3]. Given the difficulty of modeling the time-dependent nature of IFR early on in the pandemic, one interpretation of the adjusted and unadjusted models is that the true incidence is likely closer to the unadjusted model (with a higher IFR) early on and transitions to be closer to the adjusted model later on. We added text into the limitations to discuss this issue.

For the second point, thank you for pointing out the issues with a lack of uncertainty around the asymptomatic rate. The asymptomatic rate is highly uncertain, with meta-analyses point estimates ranging from 17-45% [4-6] and some other studies estimating as high as 76% [7]. The CDC still uses 40% as their best estimate. So for the adjusted method – our best guess at the true parameter values – we will still keep the 0.65% IFR and 40% asymptomatic rate. However,

for the unadjusted estimate, to give a wider range of potential values, we now use an asymptomatic rate 50% and thus a higher symptomatic case fatality rate (the table below shows the different options of IFR, asymptomatic rate, and sCFR). We also adjusted the explanation in the paper.

The limitations section in the discussion was expanded to address both of these concerns:

> *"Furthermore, mMAP and GLEAM rely on assumptions about the IFR and asymptomatic rate; point estimates of each are uncertain, with reasonable estimates ranging from 0.65% to 1.1% for the IFR and 17% to 50% for the asymptomatic rate, which therefore yields sCFR point estimates ranging from 0.78% to 2.2% (a more detailed discussion these values is provided in the mMAP methods). As well, the IFR likely evolved as the pandemic progressed because treatments were improving and different subsets of the population were infected at different times. However, there is some evidence that the IFR remained stable during the beginning of the pandemic [1,3] and a meta analysis of IFR using data until September [2] yielded similar IFR estimates to the meta-analysis using data until April [3], indicating that there is not a clear decreasing trend in IFR."*

Table Demonstrating Different IFR, Asymptomatic Rate and sCFR Scenarios.

Method	*Infection Fatality Rate (%)*	*Proportion Asymptomatic*	*Symptomatic Case Fatality Rate (%)*
Unadjusted (currently)	*1.1*	*0.4*	*1.8*
Adjusted (currently)	*0.65*	*0.4*	*1.1*
Unadjusted v1	*1.1*	*0.2*	*1.4*
Unadjusted v2	*1.1*	*0.5*	*2.2*
Unadjusted v2	*1.1*	*0.76*	*4.6*

[1] Hauser, Anthony, et al. "Estimation of SARS-CoV-2 mortality during the early stages of an epidemic: a modelling study in Hubei, China and northern Italy." medRxiv (2020).

[2] Levin, Andrew T., Kensington B. Cochran, and Seamus P. Walsh. Assessing the age specificity of infection fatality rates for COVID-19: Meta-analysis & public policy implications. No. w27597. National Bureau of Economic Research, 2020.

[3] Meyerowitz-Katz, Gideon, and Lea Merone. "A systematic review and meta-analysis of published research data on COVID-19 infection-fatality rates." medRxiv (2020).

[4] Byambasuren, Oyungerel, et al. "Estimating the extent of true asymptomatic COVID-19 and its potential for community transmission: systematic review and meta-analysis." Available at SSRN 3586675 (2020).

[5] Buitrago-Garcia, Diana C., et al. "The role of asymptomatic SARS-CoV-2 infections: rapid living systematic review and meta-analysis." medRxiv (2020).

[6] Oran, Daniel P., and Eric J. Topol. "Prevalence of Asymptomatic SARS-CoV-2 Infection: A Narrative Review." Annals of Internal Medicine (2020).

[7] Poletti, Piero, et al. "Probability of symptoms and critical disease after SARS-CoV-2 infection." arXiv preprint arXiv:2006.08471 (2020).

2) Why only focus on excess pneumonia deaths to adjust for underreporting of COVID-19 deaths? I believe that the CDC provides state-level data on all-cause mortality compared to COVID-19 mortality (https://www.cdc.gov/nchs/nvss/vsrr/covid19/excess_deaths.htm#data-tables), and these estimates could be used to provide an upper bound on true deaths for the whole dataset.

Response: It is true that there has been a noticeable increase in mortality from causes other than pneumonia, but we chose to not include these for two reasons: (1) fatality rates were not usually calculated using excess deaths and (2) many of these excess deaths are likely not due to direct causes of COVID-19.

The fatality ratios used in this study were calculated by using confirmed or probable COVID-19 deaths; the "unadjusted" fatality ratio calculated from a line list of COVID-19 cases and deaths [1] and the "adjusted" fatality ratio calculated from a meta-analysis of studies using reported death counts [2]. Therefore, the numerator of the assumed IFR does not include excess or unreported COVID-19 deaths. The numerator of the assumed IFR should match the deaths calculated in this study since we roughly calculate cases = deaths/IFR. While the COVID-19 line list used to calculate the first, higher IFR of ~ 1.1% might have better captured COVID-19 deaths and thus fewer misattributed/unreported deaths, this would lead to a higher IFR estimate and lower estimate of cases by mMAP. This issue of death reporting points to the fact that including excess influenza and pneumonia deaths in COVID-19 death counts could result in an overestimate of COVID-19 cases, though we still think it is reasonable to include them as an upper bound.

As well, it is difficult to determine how much of the excess deaths are directly caused by COVID-19 compared to random yearly fluctuations or indirect causes of COVID-19. Aside from the IFR issue discussed, in this study we chose to only study excess deaths that are directly caused by COVID-19 as opposed to indirect causes such as worse hospital treatment and reduced access to healthcare facilities. As an example, of the CDC lists of excess deaths caused by major diseases, 30% of excess deaths by April 4, 2020 and 34% of excess deaths by May 16, 2020 are caused by Alzheimer's Disease and Dementia [3], though it is unclear how

many of these deaths are due to unreported COVID-19 as opposed to reduced quality of care, isolation, or other reasons.

While excess deaths are an important statistic for evaluating the burden of COVID-19 on the population and the progression of the impact of the epidemic, we do not feel that they are the right statistic to use for estimating cases from the mMAP method.

[1] Verity, Robert, et al. "Estimates of the severity of coronavirus disease 2019: a model-based analysis." The Lancet infectious diseases (2020).

[2] Meyerowitz-Katz, Gideon, and Lea Merone. "A systematic review and meta-analysis of published research data on COVID-19 infection-fatality rates." medRxiv (2020).

[3] Centers for Disease Control and Prevention. "Weekly counts of death by jurisdiction and cause of death." Provisional death counts for coronavirus disease (COVID-19) (2020). https://data.cdc.gov/NCHS/Weekly-counts-of-death-by-jurisdiction-and-cause-o/u6jv-9ijr/ visited on 11/2/2020.

3) Is the COVID scaling approach fair? Even after accounting for sparse case counts, testing backlogs, and false negatives, I find the assumption of uniformly applied testing problematic (after line 431—line numbering seems to have gone awry in this section). Testing shortages, especially early in the epidemic, may have biased testing toward higher-risk subjects. As testing capacity increased across states, test positivity may have gone down simply due to testing more people, while true prevalence (and therefore, presumably fraction of ILI infected with COVID) remained relatively constant. While this limitation is mentioned in lines 254-255, I think that additional discussion is needed, as this assumption of unbiased testing is almost certainly broken.

That is an excellent point. Our baseline assumption of uniform testing is that because our population consists of patients with ILI symptoms, then conditional on having ILI symptoms, the probability of getting tested is independent of whether the patient has COVID-19 or some other infection. However, other factors such as prior likelihood of exposure could bias testing. Because this form of confounding is difficult to measure, we have conducted a sensitivity analysis using a probabilistic model and added the details and results in the Appendix. As an example, when COVID-19 positive patients are 25% more likely to get tested, our methods show that the median state's true case count is 89% of what is estimated by COVID scaling. When 50% more likely to get tested, the resulting median true count is 81% of what is estimated.

We added the following to the discussion to emphasize the point:

"This assumption may be problematic when prior suspicion of exposure is involved, such as when health workers at a nursing home outbreak are preemptively tested, and may be a greater issue during testing shortages. In a sensitivity analysis, we computed the hypothetical impact of testing bias, finding that in the most extreme case, the true case count could be 80% of what we estimated (Appendix 5)."

We added the following to the methods:

"The uniform testing assumption relies on the premise that conditional on having ILI symptoms, the probability of getting tested is independent of whether the patient has COVID-19 or some other infection. This assumption is likely inexact when additional factors such as prior exposure caused biased testing towards patients more likely to be COVID-19 positive. While the impact of these factors cannot be measured in our data, we conduct a sensitivity analysis in Appendix 5 that models testing bias during low test availability to assess their potential impact on our estimates."

Minor points:

1) I think it would help with clarity to provide the names for the three approaches (divergence, scaling, and mortality) in the introduction.

Response:

We appreciate the helpful suggestion and agree it improves clarity. We have added the names in the introduction, for example:

"Importantly, regional increases in ILI observed from February to April 2020 in conjunction [...] We denote such methods as the divergence *approach.*

A second and related approach (denoted as COVID Scaling*) [...]*

Our third approach (termed mMAP*) uses [...]"*

2) Line 282: "(up to 4%) of the US population may have already been infected." It would be good to specify the date here, since I assume that this was using the estimates of true infections at the end of May. In that case, the following statement that "subsequent waves of infection may decrease in magnitude" should be revaluated in the context of extant case data for the second wave of infection that occurred during the summer.

Response: *We would like to thank the reviewer for this point. We added the date, and in light of more recent information, we have changed the language in our conclusion:*

"A more accurate picture of the burden of COVID-19 is actionable knowledge that will help guide and focus public health responses. As social distancing measures are being (or have been) relaxed, some locations are experiencing a resurgence in cases. If the true case counts are near the upper bound of our estimated symptomatic case count, then a substantial proportion (up to 3% as of May 16) of the US population may have already been infected. Factoring in asymptomatic cases this could increase the proportion up to 8%. On the other hand, it is evident that the large majority of the population has not yet been exposed to COVID-19, and therefore effective, informed public health responses to future upsurges in cases will be essential in the upcoming months."

3) Line 353: I think a reference to either the supplement or previous section is missing.

Response: *Thank you for catching that. We have fixed the reference in our manuscript*

4) It seems that the mortality method assumes that deaths are reported on the actual date of death. It is not clear that all states report deaths in this way, and I am curious if accounting for delays in death reporting could be incorporated into the MAP framework.

Response: *Thank you for pointing out the issue with reporting of deaths and how it might affect our estimates. To address this issue, we added a paragraph in the discussion section about the limitations of mMAP and why we did not shift it for the delay in death reporting:*

"As well, the data used and delays in reporting affect the timing of the methods' estimates. The Divergence and COVID scaling methods estimate the date of medical visitation for ILI/COVID-19 symptoms while the mMAP and GLEAM methods estimate the date of COVID-19 symptom onset, which is expected to be on average 4-5 days before medical visitation [reference Li Early Transmission]. However, mMAP and GLEAM estimates are shifted later by the delay in death reporting, likely making the dates of estimation of the methods fairly close. There is limited research on quantifying this delay, though one study found it to be 4.29 days for Mexico and 1.74 for England, with a wide range heterogeneity between localities [reference Gutierrez Delays in Death Reports]."

5) References 70 and 71 seem to be misformatted.

Response: Thank you for pointing that out. We have now fixed the formatting for these references, though we now use a different reference for the CDC excess death data since the CDC updated their method of reporting excess deaths.

6) Supplement, after equation 7 should "P(t=0)" be "p(t=0)?" I didn't quite follow what the change in summation limits here was.

Response: Thank you for noticing the improper notation in the supplementary materials. In our second submission, the supplementary materials for mMAP have been adjusted based on improvements in the method. The stopping criteria for the iterations in mMAP was updated to be based on a chi-squared stopping criteria (this information is now provided in the methods section) as opposed to a relative difference stopping criteria which was done before. We changed this because the chi-squared criteria limits overfitting to the noise in the death data, which is especially an issue with mMAP since there are as many unknown parameters (cases) as observed data points (deaths). Previous work on deconvolution for the 1918 flu pandemic recommended this stopping criteria [1]. The mMAP predictions using this stopping criteria appear to be more realistic and robust (fewer dramatic spikes in cases that seem unrealistic).

Given this new stopping criteria, we won't have complete convergence in the end, as in we won't have the case time series in the final step exactly equal to the case time series in the step before. Therefore, the previous supplementary sections 4.1 and 4.2 do not hold for mMAP so

we removed them. We added in another section to demonstrate how mMAP is similar to the Richardson-Lucy method used in [1] and in positron emission tomography, and reference work that provides more mathematical justification for these methods.

[1] Goldstein, Edward, et al. "Reconstructing influenza incidence by deconvolution of daily mortality time series." Proceedings of the National Academy of Sciences 106.51 (2009): 21825-21829.

[1] Wu, S.L., Mertens, A.N., Crider, Y.S. et al. Substantial underestimation of SARS-CoV-2 infection in the United States. Nat Commun 11, 4507 (2020).
https://doi.org/10.1038/s41467-020-18272-4

Have all data underlying the figures and results presented in the manuscript been provided?

Large-scale datasets should be made available via a public repository as described in the PLOS Computational Biology data availability policy, and numerical data that underlies graphs or summary statistics should be provided in spreadsheet form as supporting information.

Reviewer #1: ***No:*** *The authors state that data will be made available upon acceptance.*

Reviewer #2: ***No:*** *Authors have said that data will be provided in a repository upon acceptance. It is not currently available.*

Reasoning for the addition of GLEAM:

Since our first submission, we added an additional modeling method, the Global Epidemic and Mobility model (GLEAM). Unlike the other approaches, GLEAM is a mechanistic metapopulation model that simulates the transmission of SARS-CoV-2 using large scale in-silico simulations. While mechanistic models require more information on the disease transmission process, need high-resolution datasets on the location and mobility of populations, and make assumptions about how individuals interact, they provide valuable insight into the spatiotemporal spread and magnitude of epidemics and are frequently used to model the COVID-19 pandemic. This type of modeling also incorporates information on behavioral changes such as a change in mobility or contact patterns due to interventions/government restrictions (ex. school closures and stay at home orders). Our results show that while the underlying model dynamics are different across the 4 approaches, we find a good agreement between these models. We believe that the addition of GLEAM increases the robustness of the claims in the paper by further diversifying our modeling approaches and not altering the final conclusions.
