# Supplementary material for: Estimating the cumulative incidence of COVID-19 in the United States using influenza surveillance, virologic testing, and mortality data: Four complementary approaches
Source: PLoS Comput Biol. 2021 Jun 17;17(6):e1008994. doi: 10.1371/journal.pcbi.1008994 (PMC8241061; doi:10.1371/journal.pcbi.1008994)
Supplement: Attachment — Submitted filename: March PLOS point by point responses.docx [file pcbi.1008994.s008.docx]

Dear Editor,

Thank you for the opportunity to address the additional questions from the reviewers. We appreciate the points brought up and believe our resulting modifications to the text improve the clarity of our methods. Following are our point-by-point responses:

Comments to the Authors:

1) Line 619: "We assume varying levels of effectiveness of the mitigation policies...":

It was unclear to me how this was accomplished. Does this mean that effectiveness varied based on which policies different states adopted, or did the same policy adopted in two different states have different effectiveness?

We apologize for the lack of clarity on that point. We have now added details on the implementation of the policies in the text detailing the model as it follows:

"As our model considers contact matrices for different settings, namely households, schools, workplaces and community contacts \cite{mistry2020inferring, prem2017projecting}, we quantify the decrease in contacts that individuals have in each of these environments. To implement school closures in the United States we follow~\cite{de2018impact} where authors study the effects of school closure in the context of seasonal influenza epidemics. According to the date when schools were closed in the different stateswe consider a reduction of contacts in all individuals attending an educational institution~\cite{NYT, Oxford}. This intervention was applied at state level. Following the school closure, most US states issued a ``stay at home order". In this case, we consider that only contacts in the household and essential workplaces were available. Using the COVID-19 Community Mobility reports~\cite{google_data} we compute the relative reduction on the number of contacts in workplaces, and community interaction as well as the relative reduction in the intra-country mobility. When the interventions are relaxed the mobility reduction is relaxed accordingly. From the Google mobility reports we use the field \texttt{workplaces percent change from baseline} to infer contacts reduction in workplaces, the average of the fields \texttt{retail and recreation percent change from baseline} and \texttt{transit stations percent change from baseline} for the general community settings. The Google mobility report provides the percentage change $r_l(t)$ on day $t$ of total visitors to specific locations $s$ with respect to a pre-pandemic baseline. We turn this quantity into a rescaling factor for contacts such as $\omega_s(t)=\omega_s (1 + r_l(t) / 100)^{2}$, by considering that the number of potential contacts per location scales as the square of the the number of visitors. Finally in each state we explore different levels of overall transmissibility reduction (0-30\%, step 10\%) due to the awareness of population and behavioral changes starting at the date of the state of the emergency in the US. "

2) Line 621: "We then perform model selection...": This is related to my previous point, but I think more clarity is needed regarding what precise models were compared.

We thank the reviewer for this point. We added more details on this point along with the discussion of the previous point. namely:

"By using the global calibration we generate an ensemble of epidemic models defined by the posterior distribution of the parameters and the interventions in each state that provides the weekly number number of new deaths by using available estimates of infection fatality rate ~\cite{CDC2020covidplanning,wu2020estimating,verity2020estimates}. For each model that satisfies the global calibration, we use the Akaike Information Criterion (AIC) with information loss $\Delta_i<9$. The selected models define the median and 95\% CI for cumulative infections in each state (Fig. \ref{fig.AR})."

3) Are the specific assumptions about the prior distribution of key parameters for the U.S. model available (e.g., incubation period, IFR, duration of infectiousness, etc.)? Are they the same as the assumptions made for the global calibration of the model [1]?

Yes, we use the same prior distributions of the reference cited by the reviewer. We now clarify this in the text.

4) Does the model's assumption of no pre-symptomatic transmission affect the estimates of the true number of infections? This might be useful to add to Table 2, or as a point in the discussion of limitations.

The pre-symptomatic transmission stage is included in the infectious period of individuals and not modeled explicitly. We now specify this in the text and in the Table 2.
